# Fe(II) reduction of pyrolusite (β-MnO_2_) and secondary mineral evolution

**DOI:** 10.1186/s12932-017-0045-0

**Published:** 2017-12-05

**Authors:** Michael V. Schaefer, Robert M. Handler, Michelle M. Scherer

**Affiliations:** 1 Environmental Sciences, University of California, Riverside, CA 92521 USA; 2 Sustainable Futures Institute, Michigan Technological University, Houghton, MI 49931 USA; 3 Civil and Environmental Engineering, University of Iowa, Iowa City, IA 52242 USA

**Keywords:** Manganese oxide, Fe(II), Redox, Electron transfer, Mössbauer spectroscopy

## Abstract

Iron (Fe) and manganese (Mn) are the two most common redox-active elements in the Earth’s crust and are well known to influence mineral formation and dissolution, trace metal sequestration, and contaminant transformations in soils and sediments. Here, we characterized the reaction of aqueous Fe(II) with pyrolusite (β-MnO_2_) using electron microscopy, X-ray diffraction, aqueous Fe and Mn analyses, and ^57^Fe Mössbauer spectroscopy. We reacted pyrolusite solids repeatedly with 3 mM Fe(II) at pH 7.5 to evaluate whether electron transfer occurs and to track the evolving reactivity of the Mn/Fe solids. We used Fe isotopes (56 and 57) in conjunction with ^57^Fe Mössbauer spectroscopy to isolate oxidation of Fe(II) by Fe(III) precipitates or pyrolusite. Using these complementary techniques, we determined that Fe(II) is initially oxidized by pyrolusite and that lepidocrocite is the dominant Fe oxidation product. Additional Fe(II) exposures result in an increasing proportion of magnetite on the pyrolusite surface. Over a series of nine 3 mM Fe(II) additions, Fe(II) continued to be oxidized by the Mn/Fe particles suggesting that Mn/Fe phases are not fully passivated and remain redox active even after extensive surface coverage by Fe(III) oxides. Interestingly, the initial Fe(III) oxide precipitates became further reduced as Fe(II) was added and additional Mn was released into solution suggesting that both the Fe oxide coating and underlying Mn phase continue to participate in redox reactions when freshly exposed to Fe(II). Our findings indicate that Fe and Mn chemistry is influenced by sustained reactions of Fe(II) with Mn/Fe oxides.

**Electronic supplementary material:**

The online version of this article (10.1186/s12932-017-0045-0) contains supplementary material, which is available to authorized users.

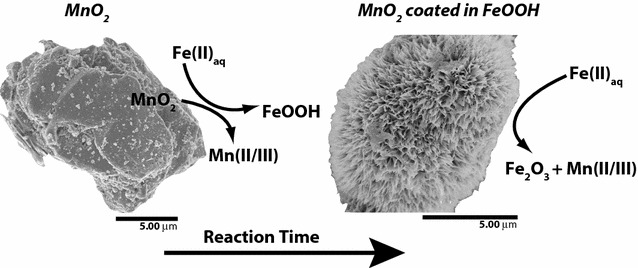

## Introduction

Iron (Fe) and manganese (Mn) are the two most common redox-active elements in the Earth’s crust [1]. Reactions between Fe and Mn species, as well as with other common groundwater constituents, have significant impacts on mineral formation and dissolution [2], trace metal sequestration [3], and contaminant transformations [4, 5]. Our understanding of the health effects of Mn exposure to humans is also evolving, and recent research indicates that elevated Mn concentrations in drinking water may lead to developmental disorders in children, among other adverse health effects [6–9]. The present study focuses on redox reactions of ferrous iron (Fe(II)) with oxidized Mn(IV) solids at circumneutral pH. Thermodynamics predict that in the presence of Fe(II), all manganese species would exist as reduced Mn(II) as opposed to oxidized Mn(IV). Complex environmental systems, however, do not always adhere to the compositions implied by thermodynamic constraints, especially in complex media such as soil aggregates [10]. For example, microorganisms can significantly impact the speciation of Fe and Mn between reduced and oxidized forms [11, 12] and lead to high local concentrations of dissolved Fe(II) or to Mn(IV) solids that form and persist in the presence of Fe(II) on transient but relevant time scales.

Certain geochemical conditions (low pH, anaerobic zones, presence of organic matter) can stabilize aqueous Fe(II), allowing reduced Fe to travel significant distances and interact with a variety of mineral species. Examples of scenarios where geochemical and kinetic effects dictate the redox interactions observed in field settings are widespread. Studies of both freshwater and marine porewater constituents have observed dissolved Fe(II) in the presence of Mn oxides [13] and implicated Mn oxides as the relevant oxidants of Fe(II) in such systems [14]. Interactions between Fe(II) and Mn oxides have been studied previously under several model geochemical settings, including acid mine drainage [15–17] and marine systems [14]. The reaction of Fe(II) with Mn(IV) oxides has previously been described [18] by reaction 1, where Fe(II) is oxidized to Fe(III) with a coupled complete reduction of Mn(IV) to Mn(II).

1 $$2 {\text{Fe}}^{ 2+ } + {\text{ MnO}}_{ 2} + {\text{ 2H}}_{ 2} {\text{O}} \to 2 {\text{FeOOH }} + {\text{ Mn}}^{ 2+ } + {\text{ 2H}}^{ + }$$

This reaction was proposed in order to explain locally high concentrations of Mn oxides (e.g. formation of Mn nodules) in sediments [18]. Redox reactions of Fe(II) with Mn(IV) oxides result in an oxidized Fe species, which often occurs as a surface coating on the underlying Mn oxide substrate. The composition of resulting Fe(III) oxide(s) has previously been difficult to ascertain with traditional methods of solid-phase analysis such as X-ray diffraction (XRD) or electron microscopy (Table 1). Postma [18] was unable to clearly define the Fe oxide coating that occurred on birnessite (δ-MnO_2_) particles reacted with Fe(II), and in later studies chose to model the resulting Fe(III) oxide phase as an amorphous Fe(OH)_3_ species while identifying it as 6-line ferrihydrite based on XRD [19]. Krishnamurti et al. [20] used a combination of infrared spectroscopy, XRD, and transmission electron microscopy (TEM) to determine that Fe(II) in contact with different Mn oxide substrates (e.g., cryptomelane, hausmannite, and pyrolusite) will react to form different Fe oxide precipitates (akaganeite, magnetite, and lepidocrocite), depending on solution conditions (Table 1).

**Table 1:** Summary of experimental results of previous studies of Fe(II) reacted with Mn-oxides

Mn-oxide substrate	Fe-oxide formed	Conditions	Notes	References
Birnessite [δ-MnO_2_]	FeOOH	pH 3–6	Fe(III) stays in solution for pH < 4 and XRD inconclusive	[18]
Birnessite [δ-MnO_2_]	Lepidocrocite and trace goethite	pH 4–6	Higher pH produces more goethite, noncrystalline Fe-oxide	[32]
Hexagonal birnessite	Lepidocrocite and goethite	pH 4–7	Less goethite under anoxic conditions compared to oxic	[48]
Cryptomelane [KMn_8_O_16_]	Akaganeite-FeOOH	pH 3–6	–	[20]
Hausmannite [Mn_3_O_4_]	Magnetite Fe_3_O_4_	pH 3–6	–	[20]
MnO_2_	Fe(OH)_3_	–	–	[15]
MnO_2_	Fe(OH)_3_; 6-line ferrihydrite	Column pH 2.5–6	Natural Mn-oxide coated sand	[19]
Pyrolusite [β-MnO_2_]	Fe-oxyhydroxide, lepidocrocite	pH 3–6	–	[20]
Pyrolusite coated silica sand	2-line ferrihydrite and jacobsite (MnFe_2_O_4_)	pH 3	Fe(III) precipitates inhibit reductive dissolution of pyrolusite by Fe(II)	[16]
Pyrolusite coated quartz	Schwertmannite or sulfate-substituted ferrihydrite	pH 3	–	[17]
Poorly crystalline MnO_2_ (similar to birnessite) and freshwater sediment	Amorphic Fe(III) oxide	pH 7	–	[49]
Vernadite [δ-MnO_2_]	Fe_2_O_3_	pH 7.4	Fe phase proposed in equation but not characterized	[50]

Formation of an Fe(III) surface coating on Mn oxide solids may impact the rate or overall ability of Mn oxides to remain redox-active phases in environmental systems. In simulated acid-mine drainage systems, Mn(II) production from Mn oxides reacted with Fe(II) decreases with time, suggesting that evolution of a new Fe oxide surface interferes with the ability of underlying Mn oxides to accept electrons from aqueous Fe(II) by creating a passivating Fe-oxide layer [16]. Further studies in this experimental system attributed changing rates of Fe(II) loss and Mn(II) production from batch reactors to Langmuir-type blocking of Mn(IV) surface sites by Fe(III) oxide precipitates using model simulations [17]. In these studies, it was also difficult to concretely ascertain the composition of resulting Fe(III) reaction products. Fe(II)/Mn(IV) redox activity may decrease the oxidation capacity of Mn oxides, which have been demonstrated to be important oxidants for a variety of environmental processes including abiotic release of organic nitrogen in soil [21] and contaminant remediation processes [22, 23]; formation of an amorphous Fe(III) precipitate has previously been shown to inhibit Cr(III) oxidation by birnessite at pH 5.5 [24].

Our guiding hypothesis was that precipitation of Fe(III) minerals at the Mn oxide surface would lead to partial passivation of the Mn oxide reactivity. Thus, we evaluated the effect of aqueous Fe(II) on electron transfer reactions at Mn oxide surfaces by subjecting pyrolusite to successive exposures of Fe(II) at pH 7.5. Pyrolusite was chosen as a model Mn-oxide for this study because it is the most thermodynamically stable Mn mineral phase, and therefore represents the end-member case for Mn(IV) reduction by Fe(II). Many investigations involving Fe(II) and Mn oxides have occurred at lower solution pH values between 3–6 in order to simulate acid mine drainage conditions. Evaluation of Fe/Mn redox chemistry at circum-neutral pH values is also important, as anoxic Fe(II) plumes may persist in neutral pH environments in the presence of Mn oxides [25].

Alongside traditional methods of analysis (XRD, scanning electron microscopy, chemical Fe and Mn analyses), we utilized ^57^Fe Mössbauer spectroscopy in conjunction with isotopically enriched ^57^Fe(II) in order to increase the Fe signal (natural Fe contains ~ 2.2 mol% ^57^Fe). To further examine Fe(III) surface precipitate morphology and what effect this phase has on subsequent redox reactions with Fe(II), we exposed Mn oxide particles to a series of solutions buffered at pH 7.5 which contained either ^57^Fe(II) (Mössbauer-visible) or ^56^Fe(II) (Mössbauer-transparent). In this manner, we could subject Mn oxide solids to a series of Fe(II) exposures, but only a particular “pulse” of Fe(II) would be visible with Mössbauer spectroscopy throughout the experiment. Isotope labeling allowed us to track the chemical changes that occurred to a specific set of Fe atoms, even as more Fe(II) was introduced to the reactor.

## Experimental

### Mn oxide solids characterization

**Fig. 1:**
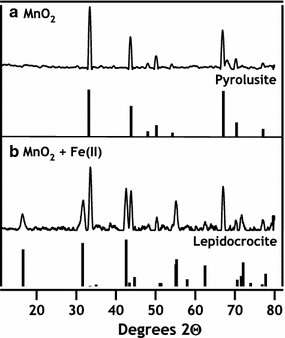
X-ray diffraction patterns (Cu Kα) of MnO_2_ particles before (**a**) and after (**b**) reaction with aqueous Fe(II). Pyrolusite and lepidocrocite standard diffraction patterns are provided for reference

Commercially available MnO_2_ (Sigma-Aldrich) was used for the entirety of the present study. Mn oxide was ground with a mortar and pestle before sieving (150-µm mesh) to achieve a uniform particle size. X-ray diffraction (XRD) was performed on prepared solids using a Rigaku Miniflex II equipped with a Co X-ray source and indicated pyrolusite (β-MnO_2_) was the sole Mn oxide phase, and no diffraction peaks indicative of impurities could be detected (Fig. 1). Surface area measurements on sieved Mn oxide powders were made with a Quantachrome BET Nova surface area analyzer using a multipoint measurement and consistently resulted in specific surface area measurements of 1–2 m^2^ g^−1^.

### Sequential batch experiments with isotopically-enriched aqueous Fe(II)

All reagents were used as received. Experiments were performed in an anoxic chamber with a 95% N_2_, 5% H_2_ atmosphere. The chamber contained multiple palladium catalysts to scavenge trace O_2_ and maintained an O_2_ level below 1 ppmv. All solutions were made with deionized water (> 18.2 MΩ-cm) that had been deoxygenated by N_2_ sparging and degassing in the anaerobic chamber. Aqueous Fe(II) stock solutions were prepared by dissolving enriched ^56^Fe or ^57^Fe metal (Chemgas, 99 and 96%, respectively) in 0.5 M HCl [26]. To initiate Fe(II) redox experiments, 18 mL of a pH 7.5 buffer solution [25 mM 4-(2-hydroxyethyl)-1-piperazineethanesulfonic acid (HEPES) + 25 mM KBr] was spiked with either a ^57^Fe or ^56^Fe stock solution to yield an initial aqueous Fe concentration of approximately 3 mM. Prior to Fe addition, reactors were counter-spiked with an equivalent volume of 0.5 M NaOH to maintain initial pH. Reactors were equilibrated for 1 h before filtering through a 0.2-µm syringe filter to remove any potential Fe precipitates resulting from trace oxidants. Initial Fe(II) concentration was then measured, and 18 mg of pyrolusite was added to initiate the experiment (solids loading 1 g L^−1^, Fe/Mn molar ratio ~ 0.26). Reactors were placed on an end-over-end rotator and mixed in the dark. Periodically, small aliquots (~ 150 µL) of suspension were withdrawn, filtered with 0.2-µm nylon syringe filters, and used for chemical Fe and Mn analyses. Experiments were typically allowed to run for ~ 90 min. If solids for a particular experiment were scheduled to receive more than 1 treatment in an aqueous solution, experimental reactors were allowed to stand for a short amount of time to allow Mn solids to settle, where they could be easily removed with a pipette. Solids were placed in a microcentrifuge tube and centrifuged inside the anoxic chamber to pellet solids and facilitate removal of the residual aqueous supernatant. Mn solids were then resuspended in a new buffer solution containing 3 mM ^57^Fe(II), ^56^Fe(II), or no Fe, depending on the particular experiment, and an additional experiment was performed to investigate the movement of aqueous Fe and Mn into or out of solution. Solids were resuspended in new buffer solutions with or without additional aqueous Fe(II) from 1 to 9 times.

### Acid extractions

To reconcile the amount of Fe(II) lost from solution with the production of Mn(II) into solution, acid extractions were performed on recovered solids to measure total Fe and Mn species. Control reactors with an identical buffer system, Mn solids loading, and Fe/Mn ratio were mixed for 90 min before solids were collected and resuspended in deionized water. 5 M HCl was added to different reactors to obtain a distribution of pH values between ~ 1–2. Extraction reactors were allowed to mix for ~ 150–300 h, periodically removing samples for Fe and Mn analyses. Additional controls of unreacted pyrolusite in HCl resulted in no measurable Mn in solution.

### Chemical analyses

Aqueous Fe(II) was measured photometrically using 1,10-phenanthroline at 510 nm [27]. Fluoride was used to remove interferences from aqueous Fe(III) [28]. The amount of Fe(III) in solution was determined by the difference of measured Fe(II) content and the total Fe concentration measured by reducing Fe(III) to Fe(II) with hydroxylamine HCl. Aqueous Mn was determined by modifying the formaldoxime method outlined in Morgan and Stumm [29] and Abel [30] using phenanthroline to complex interfering aqueous Fe.

### Solids characterization with SEM and Mössbauer spectroscopy

At the end of each experiment solids were captured by filtration through a syringe filter with a removable 0.45-µm filter disc. A small portion of recovered solids (~ 1 mg) were removed from the filter disc and rinsed with deionized water to remove residual aqueous Fe, Mn, and buffer salts. Rinsed solids were placed on an aluminum microscopy stub and fixed with carbon tape. Imaging of resulting particles and surface precipitates was performed with a Hitachi S-4800 scanning electron microscope (SEM). Remaining Mn/Fe solids recovered after sequential reaction experiments were wrapped in Kapton oxygen-impermeable tape prior to analysis with ^57^Fe Mössbauer spectroscopy. Mössbauer spectra were collected in transmission mode using a ^57^Co source and a Janis cryostat with temperature control to 13 K. Mössbauer spectra were collected at room temperature, 140, 77, and 13 K, and data was calibrated to a spectrum of α-Fe foil collected at room temperature. Spectral fitting was performed with the Recoil Software package (http://www.isapps.ca/recoil/) [31].

## Results and discussion

### Fe(II) oxidation by Mn(IV) oxide and transformation of the secondary Fe oxide

**Fig. 2:**
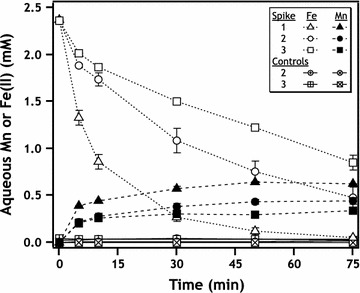
Kinetics of Fe(II) loss from (open markers) and Mn appearance into (closed markers) the aqueous phase. Triangles (Δ) indicate data for the initial suspension of pyrolusite in Fe(II), circles (○) and squares (□) indicate second and third resuspensions, respectively. Initial Fe(II) concentrations for this series of experiments were 2.4 mM. Hatched circles and squares indicate controls where Mn/Fe particles were resuspended in Fe-free buffer to check for Fe and Mn release to solution in the absence of Fe(II)

Reaction of aqueous Fe(II) with pyrolusite results in rapid loss of Fe(II) from solution and production of aqueous Mn (Fig. 2). Oxidation of Fe(II) by Mn(IV) oxide is thermodynamically favorable and well documented in the literature, although primarily under acidic pH values simulating acid mine drainage conditions [16, 18–20, 32]. At circum-neutral pH, we observed near-complete removal of 2.4 mM Fe(II) after 75 min with release of ~ 0.6 mM aqueous Mn (Fig. 2). No aqueous Mn was observed in aqueous Fe-free control experiments (Fig. 2). According to Eq. 1, loss of Fe(II) should be accompanied by formation of aqueous Mn(II) since reaction stoichiometry predicts production of half as much Mn(II) as Fe(II) oxidized [18]. Although the photometric method used to measure Mn concentrations does not permit speciation of aqueous Mn, it is reasonable to assume that aqueous Mn is most likely Mn(II) based on solubility constraints [29]. Yields of Mn release in the presence of Fe(II) are, however, lower than predicted by Eq. 1, which predicts 1.2 mM Mn released for removal of 2.4 mM Fe(II). Non-stoichiometric production of aqueous Mn has previously been attributed to adsorption or entrainment of newly-produced Mn(II) with mineral surfaces [18, 33], as well as reoxidation of Mn(II) by Mn-oxides [34].

**Fig. 3:**
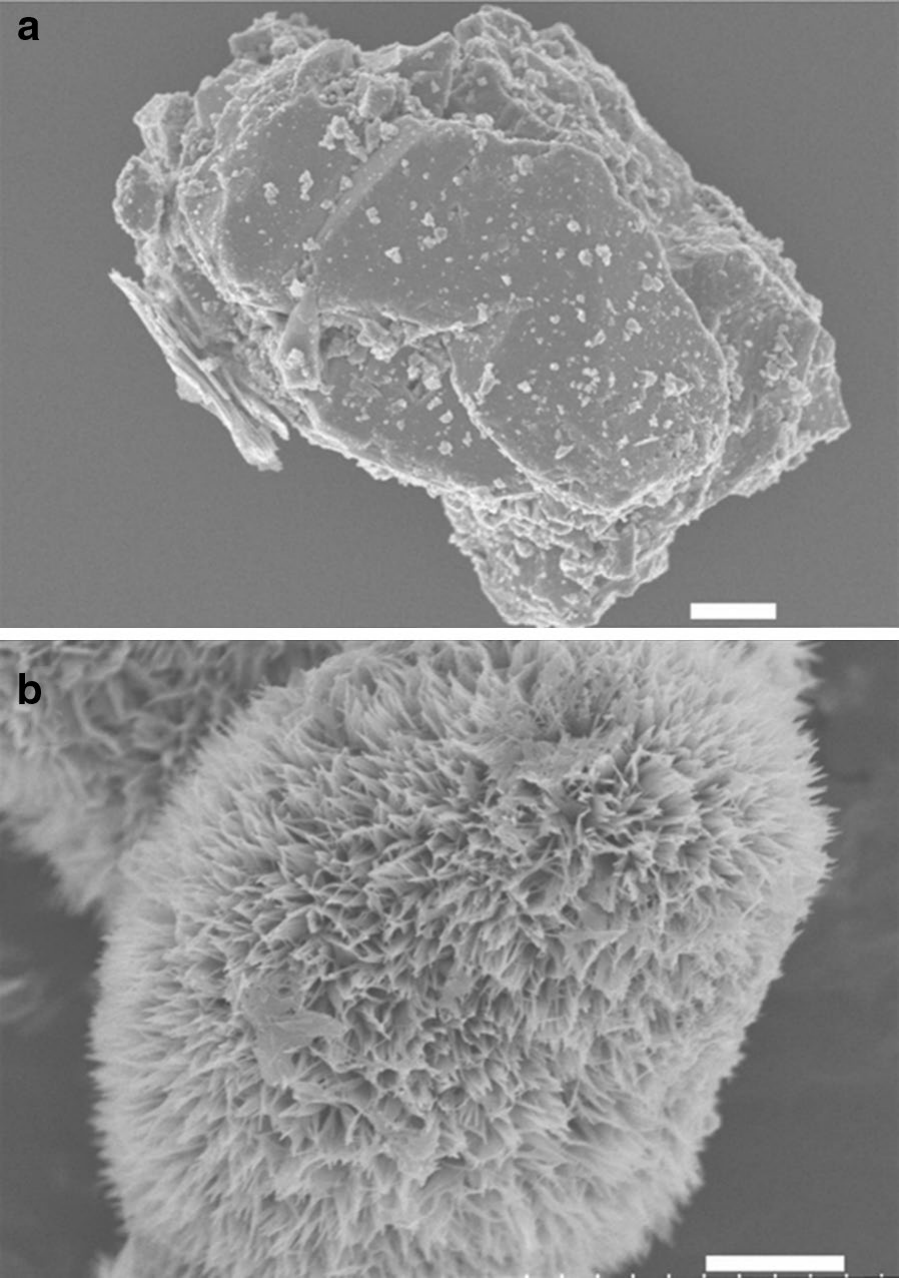
SEM images of unreacted pyrolusite (**a**) and pyrolusite reacted with 3 mM Fe(II) (**b**). The scale bar is 2 µm

**Fig. 4:**
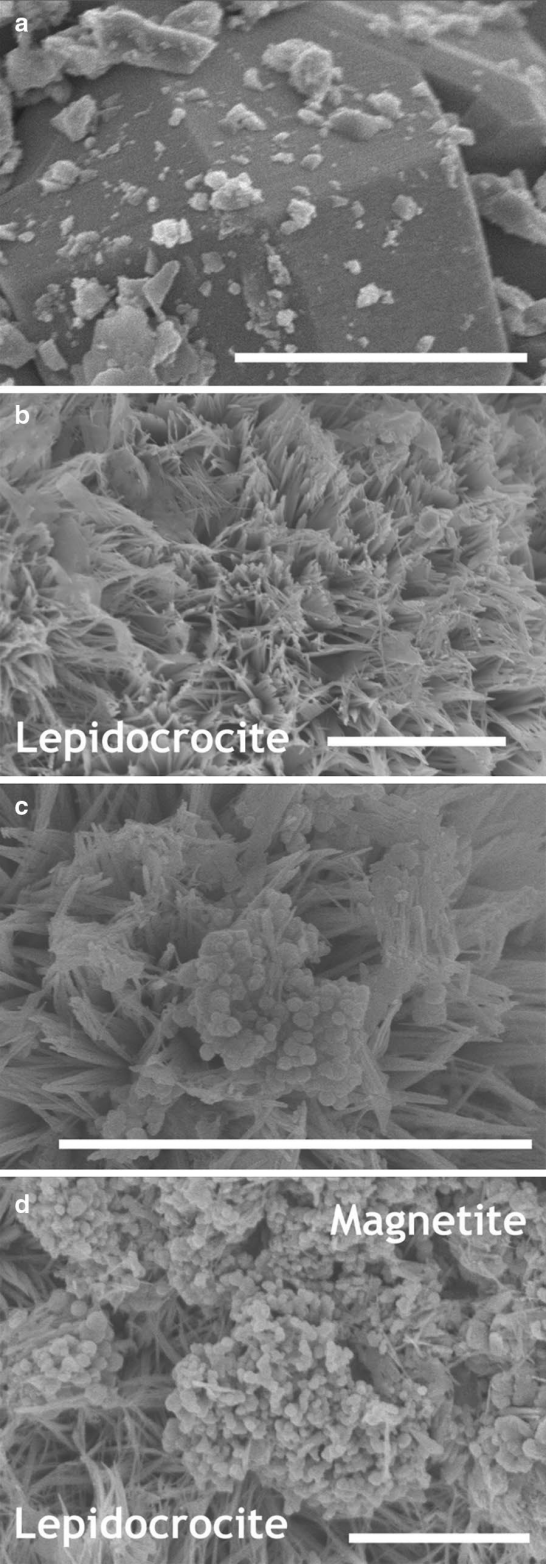
Scanning electron micrographs reveal significant changes in particle morphology that occur when unreacted pyrolusite particles (**a**) are exposed to aqueous Fe(II). After one reaction sequence of pyrolusite with 3 mM aqueous Fe(II) extensive rod-like surface precipitates (**b**) cover the surface of every particle that was imaged. Images of particles resuspended two (**c**) and three (**d**) times in 3 mM aqueous Fe(II) show a morphological transition from rod-like precipitates to spherical particles. Scale bars in all images are 2 µm

**Fig. 5:**
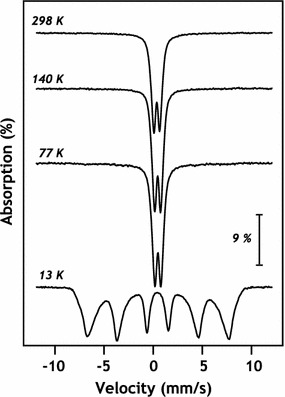
Mössbauer spectra of pyrolusite reacted with 3 mM ^57^Fe(II). Spectra were collected at temperatures ranging from room temperature (298 K) to 13 K

SEM images of pyrolusite reacted with Fe(II) show dense rod-like precipitates consistent with the oxidation of Fe(II) by pyrolusite and precipitation of an Fe(III) oxide covering the observable pyrolusite surface (Figs. 3, 4a, b). To identify the Fe(III) precipitate, solids were analyzed using powder X-ray diffraction (pXRD) and Mössbauer spectroscopy. Room temperature Mössbauer spectra collected on pyrolusite particles after reaction with Fe(II) were consistent with an Fe(III) oxide doublet with center shift (CS) 0.37 mm s^−1^ and quadrupole splitting (QS) 0.53 mm s^−1^ [35]. The Fe doublet magnetically orders into a sextet between 77 and 13 K (Fig. 5), which is characteristic of both lepidocrocite and ferrihydrite [35]. pXRD patterns of the particles after reaction with Fe(II) have reflections consistent with lepidocrocite (Fig. 1) and the rod-like morphology of the precipitates (Figs. 3, 4b) is also indicative of lepidocrocite [36].

Previous work has found that both the identity and morphology of Fe(III) precipitates formed from oxidation of Fe(II) varies depending on the Mn substrate (MnO_2_, Mn_2_O_3_, MnOOH) and solution conditions [pH, ionic strength, Fe(II) concentration] (Table 1). Most studies of Fe(II) reacting with Mn-oxides have been performed at acidic pH, while studies at circumneutral pH focused on biological mechanisms involved with the reaction. Most of these studies identified the formation of discrete Fe phases (only one study reported mixed Mn/Fe jacobsite (MnFe_2_O_4_) formation [16]) that were predominantly hydroxylated Fe phases (Fe(OH)_3_ or FeOOH) with only one study reporting magnetite (Fe_3_O_4_) formation [20]. Our observations of lepidocrocite formation from Fe(II) oxidation by pyrolusite are consistent with previous results at pH 6 where lepidocrocite was produced across a range of Mn/Fe molar ratios [20].

To probe the evolution and continued transformation of the Fe-coated pyrolusite particles, we reacted the particles with additional aqueous Fe(II). During the second and third exposure of these particles to Fe(II), Fe(II) loss from and Mn release to solution still occurred, but decreased with each exposure (Fig. 2). Images of particles taken after each Fe(II) exposure reveal changes in particle morphology from rod-like structure (1 exposure) to a mixture of rod-like and spherical structures (Fig. 2). The proportion of spherical particles increases between exposures 2 and 3, and spherical morphology is consistent with magnetite particles [36]. Magnetite formation after reaction of Fe(II) with lepidocrocite has been observed previously [37], although high sulfate concentrations (not present in our study) may inhibit this process [2, 38].

### Evolution of secondary Fe oxide

To confirm the secondary formation of magnetite or maghemite (hereafter referred to as magnetite) analysis of pyrolusite particles reacted with different sequences of isotopically-labeled Fe(II) was performed using ^57^Fe Mössbauer spectroscopy. Mössbauer spectroscopy is specific to the 57-isotope of Fe and we employed both enriched ^56^Fe(II) and ^57^Fe(II) in different reaction sequences. Iron isotope labeling allowed for the use Mössbauer spectra to track a specific set of Fe atoms (the ^57^Fe atoms) through an experiment without spectral contribution from ^56^Fe atoms. Mössbauer spectra were collected at 77 K to differentiate magnetite from lepidocrocite by minimizing errors due to superparamagnetic behavior of magnetite [39]. At 77 K, lepidocrocite [and most Fe(III) oxides] display doublet spectral features, whereas magnetite exhibits multiple sextets.

**Fig. 6:**
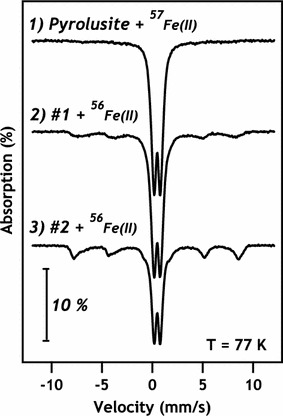
^57^Fe Mössbauer spectra of pyrolusite resuspended 1, 2, and 3 times in 3 mM aqueous Fe(II), where the Fe isotope order of addition was ^57^Fe–^56^Fe–^56^Fe. A marked increase in magnetite character can be observed in successive spectra, which is indicative of chemical transformations occurring only in the initial ^57^Fe atoms oxidized and precipitated on the pyrolusite surface. A summary of lepidocrocite and magnetite ratios obtained from fitting of the Mössbauer spectra is available in Table 2

We tracked the evolution of initially precipitated Fe atoms by beginning with a reaction between pyrolusite and ^57^Fe(II). Mössbauer spectra from this sample displayed only doublet spectral features at 77 K, characteristic of Fe(III) oxide (Fig. 6). Upon subsequent addition of ^56^Fe(II) to these solids, spectra developed sextet characteristics indicative of magnetite in addition to Fe(III) oxide doublets. The reduction of ^57^Fe(III) could result from reaction with ^56^Fe(II) via solid-state conversion of lepidocrocite to magnetite, which would result in the presence of magnetite spectral features in Mössbauer spectra. Another pathway resulting in ^57^magnetite formation is through Fe atom exchange, which has previously been observed between aqueous Fe(II) and goethite [40], magnetite [41], hematite [42], and Fe-bearing clay minerals [43]. Although direct evidence of atom exchange between lepidocrocite and aqueous Fe(II) is not yet available it likely occurs to some extent. In this pathway ^57^Fe(III) could participate in atom exchange reactions with ^56^Fe(II) and become solubilized as ^57^Fe(II), where it would be re-oxidized at the Mn(IV) or Fe(III) surface. Re-oxidation would result in precipitation of either additional lepidocrocite or magnetite through reaction of aqueous ^57^Fe(II) rather than from solid-state conversion of lepidocrocite to magnetite. Given the current data set, it is not possible to distinguish which process is the dominant pathway for magnetite formation. Most likely atom exchange reactions occur in tandem with direct Fe(II)-catalyzed lepidocrocite to magnetite conversion at the pyrolusite surface. Phase changes in Fe precipitates from oxidized lepidocrocite to mixed-valence magnetite (via net reduction of lepidocrocite) provide evidence that secondary Fe precipitates are able to participate in redox reactions with aqueous Fe(II), suggesting Mn/Fe particle complexes remain important redox-active phases in reactions with constituents like Fe(II).

**Fig. 7:**
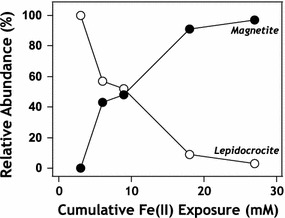
Relative abundances of lepidocrocite (open markers) and magnetite (closed markers) in marginal Fe(II) additions, as determined by Mössbauer spectral fitting of ^57^Fe phases at 77 K. Experiments were only exposed to ^57^Fe during the final Fe(II) resuspension, permitting us to view chemical changes occurring to the marginal Fe(II) addition. After initial reaction of pyrolusite with 3 mM ^57^Fe(II), only lepidocrocite was detectable in Mössbauer spectra. Increasing ^56^Fe(II) exposure prior to final ^57^Fe(II) exposure resulted in marginal ^57^Fe precipitate formation increasingly dominated by magnetite, as identified by characteristic overlapping sextets in Mössbauer spectra

**Fig. 8:**
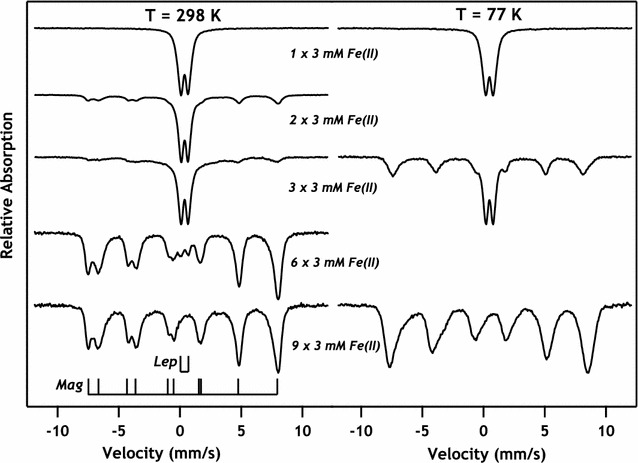
^57^Fe Mössbauer spectra of all experimental data collected in which only the final resuspension of Mn particles was done using ^57^Fe(II). Room temperature (RT, 298 K) spectra are provided for all experiments (left column), with selected 77 K spectra of identical experiments (right column) for comparison. After only one resuspension in 3 mM Fe(II), the resulting spectrum is devoid of multi-sextet character typical of magnetite. As the amount of Fe(II) exposure increases, we can see the final deposition of ^57^Fe atoms onto the particle surface results in an increasingly large multi-sextet signal and a gradual disappearance of the doublet associated with lepidocrocite formation. Comparing RT spectra with 77 K, magnetite sextets appear to overlap more thoroughly at 77 K, which is commonly observed below the Verwey transition temperature (~ 120 K). Spectra collected at 77 K also contain a visibly higher ratio of sextet: doublet spectral area, possibly indicating the presence of unordered magnetite at room temperature, which orders into a typical sextet at lower collection temperatures

Next, a similar experiment was performed except the order of Fe isotopes added to the system was switched to investigate the fate of marginal aqueous Fe(II) addition. In these experiments, initial Fe(II) additions were made with Mössbauer invisible ^56^Fe(II). Pyrolusite particles were suspended in 3 mM ^56^Fe(II) from 0 to 8 times prior to addition of ^57^Fe(II), which was always added as the final resuspension and endpoint of reaction. ^57^Fe(II) addition terminated experiments at 2, 3, 6, and 9 resuspensions [6, 9, 18, and 27 mM total Fe(II) added] and solids were preserved for Mössbauer analysis. Fits of relative peak areas in these spectra reveal that the ratio of magnetite to lepidocrocite increased with the total amount of Fe(II) added to the system, suggesting lepidocrocite was transforming to magnetite (Figs. 7, 8, Table 2). The results are consistent with previous work with hausmannite (Mn_3_O_4_) where at low Mn:Fe molar ratios lepidocrocite formation was observed while at higher Mn:Fe ratios magnetite formation occurred instead [20]. Fe(II)-induced conversion of lepidocrocite to magnetite has also been observed for pure lepidocrocite under slightly alkaline conditions similar to our experimental conditions (pH > 7.3) [37]. Under alkaline conditions the conversion of lepidocrocite to magnetite (via a green rust intermediate) in the presence of Fe(II) is rapid and complete after 10 min [44]. Sustained oxidation of Fe(II) confirms that after cumulative addition of 27 mM Fe(II), which exceeds the electron accepting capacity of original pyrolusite particles [1 g L^−1^ = 23 mM e^−^ for conversion of Mn(IV) to Mn(II)], some of the final Fe(II) added to the system was oxidized. Regardless of whether the relative oxidant in the system is Mn(IV) or Fe(III), redox reactions in Mn/Fe environments continue after the precipitation of oxide layers that have previously been considered passivating.

**Table 2:** Relative abundances of lepidocrocite and magnetite/maghemite appearing in ^57^Fe Mössbauer spectra at 77 K

Fe(II) addition	Appearance of ^57^Fe(II) in Series^a^				
	1st (Lep./Mag.)	2nd (Lep./Mag.)	3rd (Lep./Mag.)	6th (Lep./Mag.)	9th (Lep./Mag.)
# 1	100/0	NA^b^	NA	NA	NA
# 2	85/15	57/43	NA	NA	NA
# 3	75/25	46/54	52/48	NA	NA
# 6	–^c^	–	–	9/91	NA
# 9	–	–	–	–	3/97

^a^Refers to the position of single ^57^Fe(II) resuspension in the resuspension sequence. All other Fe(II) resuspensions were performed using ^56^Fe(II), which would not contribute to observed Mössbauer spectra

^b^NA due to addition of ^56^Fe(II) in this position. No Mössbauer spectral features were observed, as ^56^Fe(II) is not visible to ^57^Fe Mössbauer spectroscopy

^c^Dashed lines indicate that the experiment was not performed

### Reaction stoichiometry

**Fig. 9:**
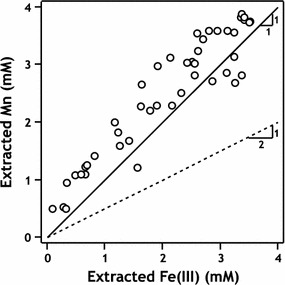
Summary of recovered Fe(III) and Mn after resuspension of Mn/Fe mixed-phase solids in HCl. Theoretical extraction results based on a 1 Fe: 1 Mn (dashed lines) or 2 Fe: 1 Mn (dotted lines) reaction stoichiometry are provided for reference. Data cluster more closely around the 1:1 reaction line, indicating that Fe(II) may be reacting with Mn(IV) to produce Mn(III), which remains in the solid phase. A majority of data points cluster above the 1:1 line, due to the presence of ~ 0.6 mM Mn already existing in solution at the onset of acid addition, as a result of the initial reaction between pyrolusite and Fe(II) (see Fig. 2). Reactions were performed in 60 mL HEPES buffer with 60 mg MnO_2_

Acid extraction (pH 1–2) of Mn/Fe particles resulted in relatively congruent dissolution of Mn and Fe(III), suggesting that Mn was evenly distributed throughout the Fe precipitate phase (Fig. 9 and Additional file 1: Table S1). Regular distribution of Mn within Fe precipitates could be evidence for cation substitution of Mn into lepidocrocite, which may also explain the low ordering temperature observed in ^57^Fe Mössbauer spectra as was previously observed with Al substitution in lepidocrocite [45]. Mn and Fe(III) dissolution occurred in an approximately 1:1 ratio rather than a 1:2 ratio predicted by Eq. 1 (Fig. 9). Mn and Fe recoveries suggest an apparent reaction stoichiometry of 1:1 between Fe(II) and Mn(IV). This suggests that the average oxidation state of acid-extractable Mn may be Mn^3+^, although Mn(III) disproportionates into Mn(II) and Mn(IV) at low pH which makes it difficult to definitely determine the reaction stoichiometry. Here we are only able to report on the average observed oxidation state of Mn based on electron balance. Control studies suspending unreacted pyrolusite particles in pH 1.0 buffered solutions, our most extreme extraction condition, did not produce detectable aqueous Mn after several days, ruling out the presence of acid-soluble Mn in the unreacted Mn(IV) solids.

**Fig. 10:**
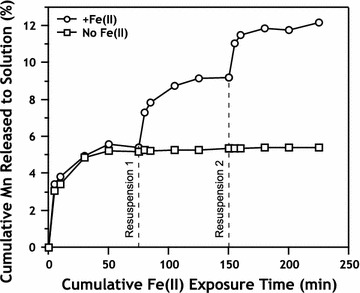
Mn released from MnO_2_ to solution after exposure to ~ 2.4 mM Fe(II), expressed as mol%. Data are plotted as cumulative reaction time, and times of resuspension at 75 and 150 min are indicated for suspension treatments with additional Fe(II) (circles) and Fe-free buffer (squares)

Resuspension of Mn/Fe particles in aqueous Fe(II) initiates a non-stoichiometric release of additional Mn to solution, but no Mn is released into solution when the particles are simply resuspended in pH 7.5 buffer without aqueous Fe(II) (Figs. 4, 10). After 3 exposures to Fe(II) and cumulative exposure time of 225 min (3 × 75-min reaction times), 13% of the initial Mn in MnO_2_ is mobilized to the aqueous phase (Fig. 10). Sustained Mn release from the solid phase to solution only in the presence of additional Fe(II) indicates further redox reactions between Fe(II) and lepidocrocite coated pyrolusite, resulting in adsorbed or structurally incorporated Mn being released during lepidocrocite transformation to magnetite. Similar release of structurally-substituted Mn from goethite and hematite in the presence of aqueous Fe(II) has been observed previously and been attributed in part to reduction of Mn [46]. Another explanation is direct interaction of Fe(II) with pyrolusite despite the presence of an Fe(III) solid layer. This could involve the conversion of lepidocrocite to magnetite and subsequent coupling of pyrolusite reduction to aqueous Fe(II) oxidation by conduction of electrons through magnetite particles. Kato et al. demonstrated magnetite’s ability to facilitate microbial interspecies electron transfer reactions [47], and we expect magnetite to also be capable of facilitating abiotic electron transfer reactions between spatially disconnected species. Regardless of the mechanism, aqueous Mn release in the presence of Fe(II) appears to occur in the presence of an oxidized Fe surface precipitate despite such precipitates generally thought to prevent Mn redox reactions by acting as a passivating layer [17, 48].

## Conclusions

Mn-oxides are powerful natural oxidants, and Mn redox cycling plays a major role in contaminant fate and transport [49]. Here we show that Fe-oxide coatings that form through the abiotic reaction of Fe(II) with Mn-oxide alter the surface properties of the Mn-oxide mineral, but do not shut down the particles’ redox activity. Our findings suggest that surface passivation through the formation of Fe-oxides may not be as extensive or complete as previously thought. In our experiments, we show that the conversion of the initially precipitated Fe-oxide (lepidocrocite) to magnetite is coincident with excess Mn release either from the underlying Mn-oxide or Mn incorporated in the lepidocrocite. These experiments were performed with pyrolusite, the most thermodynamically stable Mn(IV) oxide; thus we expect Mn-oxide reduction by Fe(II) to be a process applicable to a variety of Mn(III/IV)-oxides under environmental conditions. Our findings raise the interesting question of whether sustained redox reactivity in the presence of surface coatings is restricted to Fe(II)/Fe(III) interactions or extends to other environmentally important constituents such as reduced groundwater contaminants.

## Additional file

**Additional file 1: Table S1.** A summary of acid extraction data performed on Mn-oxides reacted with Fe(II).
