# Supplementary material for: Fe(II) reduction of pyrolusite (β-MnO2) and secondary mineral evolution
Source: Geochem Trans. 2017 Dec 5;18:7. doi: 10.1186/s12932-017-0045-0 (PMC5716966; doi:10.1186/s12932-017-0045-0)
Supplement: Supplementary file 1 — Additional file 1: Table S1. A summary of acid extraction data performed on Mn-oxides reacted with Fe(II). [file 12932_2017_45_MOESM1_ESM.docx]

**Fe(II) Reduction of Pyrolusite (ββ-MnO_2_) and Secondary Mineral Evolution**

Michael V. Schaefer^1^, Robert M. Handler^2^, and Michelle M. Scherer^3^*

^1^Environmental Sciences, University of California, Riverside, CA, 92521; mschaef@ucr.edu

^2^Sustainable Futures Institute, Michigan Technological University, Houghton, MI, 49931; rhandler@mtu.edu

^3^Civil and Environmental Engineering, University of Iowa, Iowa City, IA, 52240

*michelle-scherer@uiowa.edu

Additional file

Table S1 is a summary of acid extraction data performed on Mn-oxides reacted with Fe(II).

**Table S1**: Summary of Acid Extraction Data after Reaction of 1 g/L Pyrolusite with 3 mM Fe(II)

| **Sample No.** | **Acid Added^a^ (mmol HCl)** | **Time (hr)** | **Fe(III) Extracted (mM)** | **Mn Extracted (mM)** | **Fe(III)_ext_ : Fe(II)_0_^b^** | **Fe(III)_ext_ : Mn_ext_^c^** |
| --- | --- | --- | --- | --- | --- | --- |
| 1 | 12.5 | 24.25 | 2.51 | 3.04 | 0.74 | 0.82 |
| 2 | 12.5 | 50.50 | 3.12 | 3.59 | 0.93 | 0.87 |
| 3 | 12.5 | 98.50 | 3.35 | 3.83 | 0.99 | 0.88 |
| 4 | 12.5 | 143.75 | 3.37 | 3.87 | 1.00 | 0.87 |
|  |  |  |  |  |  |  |
| 5 | 12.5 | 24.25 | 2.43 | 3.04 | 0.72 | 0.80 |
| 6 | 12.5 | 50.50 | 2.95 | 3.59 | 0.87 | 0.82 |
| 7 | 12.5 | 98.50 | 3.42 | 3.78 | 1.01 | 0.91 |
| 8 | 12.5 | 143.75 | 3.42 | 3.83 | 1.01 | 0.89 |
|  |  |  |  |  |  |  |
| 9 | 12.5 | 24.25 | 2.55 | 3.02 | 0.75 | 0.85 |
| 10 | 12.5 | 50.50 | 3.23 | 3.55 | 0.95 | 0.91 |
| 11 | 12.5 | 98.50 | 3.52 | 3.76 | 1.04 | 0.94 |
| 12 | 12.5 | 143.75 | 3.50 | 3.73 | 1.03 | 0.94 |
|  |  |  |  |  |  |  |
| 13 | 7.5 | 7.58 | 1.23 | 1.82 | 0.34 | 0.67 |
| 14 | 7.5 | 19.42 | 1.64 | 2.66 | 0.45 | 0.62 |
| 15 | 7.5 | 77.08 | 2.59 | 3.55 | 0.71 | 0.73 |
| 16 | 7.5 | 105.58 | 2.81 | 3.59 | 0.77 | 0.78 |
| 17 | 7.5 | 213.08 | 3.24 | 3.14 | 0.89 | 1.03 |
| 18 | 7.5 | 314.50 | 3.37 | 2.81 | 0.93 | 1.20 |
|  |  |  |  |  |  |  |
| 19 | 6.25 | 7.58 | 1.42 | 1.68 | 0.42 | 0.84 |
| 20 | 6.25 | 19.42 | 1.63 | 2.28 | 0.49 | 0.71 |
| 21 | 6.25 | 77.08 | 2.61 | 3.24 | 0.78 | 0.81 |
| 22 | 6.25 | 105.58 | 2.71 | 3.44 | 0.81 | 0.79 |
| 23 | 6.25 | 213.08 | 3.12 | 2.85 | 0.93 | 1.09 |
| 24 | 6.25 | 314.50 | 3.26 | 2.69 | 0.97 | 1.21 |
|  |  |  |  |  |  |  |
| 25 | 5 | 7.58 | 0.81 | 1.41 | 0.25 | 0.58 |
| 26 | 5 | 19.42 | 1.17 | 2.00 | 0.35 | 0.58 |
| 27 | 5 | 77.08 | 1.91 | 2.97 | 0.57 | 0.64 |
| 28 | 5 | 105.58 | 2.14 | 3.13 | 0.64 | 0.68 |
| 29 | 5 | 213.08 | 2.55 | 2.81 | 0.77 | 0.91 |
| 30 | 5 | 314.50 | 2.87 | 2.71 | 0.86 | 1.06 |
|  |  |  |  |  |  |  |
| 31 | 3.75 | 7.58 | 1.56 | 1.21 | 0.46 | 1.28 |
| 32 | 3.75 | 19.42 | 1.26 | 1.59 | 0.37 | 0.79 |
| 33 | 3.75 | 77.08 | 1.78 | 2.20 | 0.53 | 0.81 |
| 34 | 3.75 | 105.58 | 1.90 | 2.29 | 0.57 | 0.83 |
| 35 | 3.75 | 213.08 | 2.16 | 2.30 | 0.64 | 0.94 |
| 36 | 3.75 | 314.50 | 2.31 | 2.51 | 0.69 | 0.92 |
|  |  |  |  |  |  |  |
| 37 | 2.5 | 7.58 | 0.34 | 0.95 | 0.10 | 0.35 |
| 38 | 2.5 | 19.42 | 0.49 | 1.08 | 0.15 | 0.45 |
| 39 | 2.5 | 77.08 | 0.66 | 1.23 | 0.20 | 0.53 |
| 40 | 2.5 | 105.58 | 0.69 | 1.26 | 0.21 | 0.55 |
| 41 | 2.5 | 213.08 | 0.66 | 1.10 | 0.20 | 0.60 |

a- Acid added as a spike of 5 M HCl.

b- Refers to the ratio of recovered Fe(III) as compared to Fe(II) concentration initially present in reactors before oxidation by pyrolusite.

c- Ratio of extracted Fe(III) to extracted Mn, at each time point.
